# Identification of Flavonoids, Antioxidant and Antiproliferative Activity of Aqueous Infusions of *Calendula officinalis* L., *Chelidonium majus* L., *Teucrium chamaedrys* L. and *Alchemilla vulgaris* L.

**DOI:** 10.17113/ftb.62.01.24.8175

**Published:** 2024-03

**Authors:** Tea Bilušić, Ivana Šola, Vedrana Čikeš Čulić

**Affiliations:** 1 Faculty of Chemistry and Technology, University of Split, Ruđera Boškovića 35, 21000 Split, Croatia; 2 Department of Biology, Faculty of Science, University of Zagreb, Horvatovac 102 a, 10000 Zagreb, Croatia; 3 School of Medicine, University of Split, Šoltanska 2, 21000 Split, Croatia

**Keywords:** antioxidant activity, antiproliferative activity, *Calendula officinalis* L., *Chelidonium majus* L., *Teucrium chamaedrys* L., *Alchemilla vulgaris* L

## Abstract

**Research background:**

The current changes in the global economy, characterised by the climate crisis and the economic and health impact of the COVID-19 pandemic, have led to a significant demand for medicinal herbs. This trend is expected to increase significantly by 2050. In this study, we investigated the biopotential of aqueous infusions of four medicinal plants: *Calendula officinalis*, *Chelidonium majus*, *Teucrium chamaedrys* and *Alchemilla vulgaris*.

**Experimental approach:**

The flavonoid analysis of the aqueous infusions of the selected plants was carried out using the RP-HPLC technique. The antiproliferative activity of the prepared aqueous plant infusions was analysed against three human cancer cell lines (MDA-MD-231, T24 and A549), while the antioxidant potential was measured using three antioxidant methods (DPPH, FRAP and Rancimat assay).

**Results and conclusions:**

*T. chamaedrys* had the highest total phenolics (expressed as GAE (2061±42) mg/L), free radical scavenging activity (IC_50_=1.9 mg/mL) and Fe(III) reducing antioxidant power (expressed as FeCl_2_ (9798±27) mg/L). At a concentration of 1 mg/mL, the antiproliferation of T24 by *C. majus* was 96 % and of MDA-MD-231 cells by *A. vulgaris* was 75 % after 72 h. After principal component analysis, *T. chamaedrys* and *C. majus* were grouped together. Quercetin glucoside and antioxidant capacity (DPPH) contributed the most to differentiate these infusions from the other two.

**Novelty and scientific contribution:**

This study represents a comparative analysis of the biopotential of four medicinal plants. A new RP-HPLC method was developed to separate the flavonoids in the herbal infusions. This is the first report on the presence of kaempferol-3-*O*-rutinoside in *C. officinalis* and isorhamnetin-3-*O*-rutinoside in *A. vulgaris* aqueous infusion. For the first time, *C. majus* has been shown to contribute to the oxidative stability of edible oil. Furthermore, this is the first comparative study on the antiproliferative activity of selected medicinal plants against the cell lines MDA-MD-231, T24 and A549.

## INTRODUCTION

According to the Market Study Report (*1*), the global market for herbal medicines is expected to reach USD 411.2 billion by 2026. A study on the use of herbal medicines in Germany found that the prevalence rate is impressively high (75.4 % 12-month prevalence, 86.7 % lifetime prevalence) (*2*). The novel coronavirus disease (COVID-19) has increased interest in immune-boosting natural preparations and the use of traditional herbal medicines (*3*). Herbal medicine includes the use of herbs, herbal materials (whole, fragmented or cut), herbal preparations and finished herbal products (*4*). In addition to their use as herbal medicines, herbal substances are also important for the production of functional foods (so-called pharma foods or medifoods or vitafoods) and as ingredients for cosmetic products. In the specialised literature, there is a new term for the interface between pharmacology and nutritional science – pharmanutrition (*5*). The field of pharmaceutical nutrition comprises the following segments: a) food supplements such as vitamins, minerals, herbs and amino acids, b) functional foods – foods that claim to improve health beyond the basic functions of nutrition, and c) medical nutrition – special food compositions for the treatment of diseases with a strong therapeutic effect (*6*).

This study focuses on screening for the biological potential (antioxidant and antiproliferative) of aqueous infusions of four medicinal plants: *Calendula officinalis* L., *Chelidonium majus* L., *Teucrium chamaedrys* L. and *Alchemilla vulgaris* L. These plant species are well documented in reports on herbal medicine. *Calendula officinalis* L. (*Asteraceae*) is known for its multiple biological activities (antioxidant, anti-inflammatory, antibacterial and antifungal) (*7*). In traditional medicine, it is used to treat wounds, ulcers, herpes, scars and skin damage (*8*). The most important flavonoids of *C. officinalis* are rutin, isorhamnetin and quercetin (*7*). Extracts from *Chelidonium majus* L. (*Papaveraceae*) show antibacterial, antiviral, antifungal and anti-inflammatory effects (*9*). The species is important in herbal medicine as a remedy for whooping cough, chronic bronchitis, asthma, gallstones, insomnia and anxiety (*10*, *11*). Derivatives of kaempferol, quercetin and isorhamnetin (kaempferol-3-*O*-rutinoside, quercetin-3-*O*-rutinoside and isorhamnetin-3-*O*-glucoside) have been identified as the most important phenolic compounds of *C. majus* (*12*). *Teucrium chamaedrys* L. (*Lamiaceae*) is used in herbal medicine for the treatment of digestive disorders, asthma, conjunctivitis and cough, as it has multiple biological effects such as antimicrobial, antiviral, antioxidant and anti-inflammatory properties (*13*). However, some authors reported the hepatotoxic effect of *T. chamaedrys* tea/decoction (*14*, *15*). The most characteristic flavonoids of *T. chamaedrys* are the flavones apigenin and luteolin (*16*). Extracts from *Alchemilla vulgaris* L. (*Rosaceae*) showed remarkable antioxidant and anti-inflammatory activities (*17*). Scientific data support its multiple biological effects (neuroprotective, gastroprotective, antimicrobial, cytotoxic and antioxidant) (*18*). The most important flavonoids in *A. vulgaris* are catechin and quercetin (*19*).

The aim of this study is to comparatively analyse the total phenolic content and dominant flavonoids in aqueous infusions of selected medicinal plants and their biological potential (antioxidant and antiproliferative). Total phenols were determined spectrophotometrically, while a new RP-HPLC method was developed to optimise the separation, tentative identification and quantification of flavonoid glycosides and aglycones. The antioxidant potential of the prepared aqueous infusions was determined using three antioxidant methods: 2,2-diphenyl-1-picrylhydrazyl (DPPH) radical assay, Fe(III) reducing antioxidant power (FRAP) assay and oxidation stability of oils and fats (Rancimat) test. The antiproliferative activity was tested on three human cancer cell lines: breast cancer cells (MDA-MB-231), bladder cancer cells (T24) and lung cancer cells (A549).

## MATERIALS AND METHODS

### Plant material and preparation of aqueous herbal infusions

The dried plant material was purchased in a phytopharmacy shop in Split (Croatia): *Calendula officinalis* L. (flower) and aerial parts of *Chelidonium majus* L., *Teucrium chamaedrys* L. and *Alchemilla vulgaris* L. The plant material was identified in the Department of Biology, at the Faculty of Science (Zagreb, Croatia). The procedure for preparing the aqueous infusion was as follows: 15 g of the plant material was infused in 200 mL of boiling distilled water for 30 min with occasional stirring, filtered through Whatman paper No. 1 and concentrated to dryness under vacuum using a rotary evaporator (Rotavapor® R-200; Büchi, Flawil, Switzerland). The obtained residue was dissolved in distilled water to a final concentration of 60 mg/mL. The prepared samples were stored at –20 °C until analysis.

### Total phenolics

Total phenolic content (TPC) was determined using the Folin-Ciocalteu reagent and the results were expressed in milligram of gallic acid equivalents (GAE) per L (*20*). Briefly, this method is based on the capacity of Folin-Ciocalteu reagent (a phosphomolybdo-tungsten hetero acid) to react with phenols and generate intense blue colour. The absorbance was recorded by Perkin-Elmer Lambda EZ 201 (Waltham, MA, USA) at 756 nm.

### RP-HPLC analysis

The Agilent 1100 Series (Agilent Technologies, Waldbronn, Germany) system was used for reversed phase-high performance liquid chromatography (RP-HPLC) analyses, equipped with the guard column Zorbax Rx-C18 (4.6 mm×12.5 mm, *d*(particle)=5 μm) and a non-polar column Poroshell 120 SB C-18 (4.6 mm×75 mm, *d*(particle)=2.7 μm). The solvents used (acetonitrile, methanol and glacial acetic acid purchased from Sigma-Aldrich GmbH, Merck (Taufkirchen, Germany) were the same as in our previous study (*21*). The profile of solvents A and B was: 100 % A and 0 % B for 0 min, 91 % A and 9 % B for 6 min, 63 % A and 37 % B for 20 min, 0 % A and 100 % B for 22 min, 0 % A and 100 % B for 27 min, and 100 % A and 0 % B for 30 min. A volume of 5 µL of each extract was injected at a flow rate of 1.0 mL/min. The column temperature was 30 °C and the absorbance was measured at *λ*=360 nm. The components were identified based on their retention times, UV spectra compared to commercial standards, and co-injections with standards. For quantification purposes, eight known concentrations of the mixed standard solution were made and calibration curves were calculated. The calibration curves are shown in Table S1 along with their R^2^ values. Results are expressed in mg/kg of plant dry mass with standard deviation (S.D.).

In order to analyse flavonoid aglycones, each extract was hydrolysed using 1.2 M HCl. The solutions were centrifuged three times using Hettich® Universal 320/320R centrifuge (Merck KGaA, Darmstadt, Germany) at 18 928×*g* for 5 min and the supernatants were stored at −20 °C until analysis.

### Antioxidant activity

The antioxidant activity of selected aqueous plant infusions was evaluated using three methods: DPPH, FRAP and Rancimat assay. The DPPH scavenging capacity of the samples was measured according to a previously described method (*22*). A volume of 50 mL of aqueous infusions of different concentrations (from 1 to 10 g/L) was placed in a cuvette with 1 mL of 6·10^–5^ M ethanolic solution of DPPH. The decrease in absorbance at 517 nm was determined by Perkin-Elmer Lambda EZ 201. The obtained results are expressed as IC_50_ values. The reducing potential (FRAP method) of the aqueous plant infusions was measured according to a previously described method (*23*). This method is based on the reduction of colourless ferric complex (Fe^3+^ tripyridyltriazine) to blue-coloured ferrous complex (Fe^2+^ tripyridyltriazine) by the action of electron-donating antioxidants at low pH. The reduction was monitored by measuring the change of absorbance at 593 nm by Perkin-Elmer Lambda EZ 201.

The effect of the infusions on the oxidative stability of extra virgin olive oil (Rancimat assay) was measured according to the described method and the results are expressed as relative protection factor (RPF) (*24*). Briefly, the induction period of the oil with and without the addition of plant infusions was determined with the Rancimat model 743 (Methrom, Herisau, Switzerland) at 120 °C and the airflow of 20 L/h. Conductivity was measured conductometrically as a function of the time.

### Cell culture

Breast cancer cells MDA-MD-231, urinary bladder cancer cells T24 and lung cancer cells A549 were purchased from ATCC (LGC Standards, Teddington, UK) and cultured for a week in a humidified atmosphere with 5 % CO_2_ at 37 °C in a Dulbecco's modified Eagle's medium (DMEM, Euroclone, Milan, Italy) containing 4.5 mg/mL glucose, 10 % foetal bovine serum (FBS) and 1 % antibiotics (penicillin and streptomycin; Euroclone).

### Cell proliferation assay

Cells were resuspended in a diluted trypan blue solution (Thermo Fisher Scientific, Waltham, MA, USA) and counted with an inverted binocular microscope (AE30; Motic, Barcelona, Spain) using the Neubauer chamber. The cell count was calculated using the following equation:

*N*(cell)=*N*(cell)_counted_·dilution·10^4^/mL /1/

Cells were then plated in 96-well plates at a density of 11 000 cell/well and incubated overnight. The cells were treated with the samples of selected aqueous plant infusions at concentrations of 0.1, 0.25, 0.5 and 1 mg/mL in triplicate for 4, 24, 48 and 72 h, respectively. Then, the 3-(4,5-dimethylthiazol-2-yl)-2,5-diphenyl tetrazolium bromide (MTT) assay was performed in such a manner that after the treatment with extracts, the cells were incubated with MTT at 0.5 g/L and 37 °C for 2 h. The medium was then removed and dimethylsulphoxide (DMSO, Sigma-Aldrich, Merck, Burlington, MA, USA) was added and incubated for a further 10 min at 37 °C with shaking. The degree of formazan formation, an indicator of living and metabolically active cells, was measured spectrophotometrically at *λ*=570 nm (HiPo MPP-96; Biosan, Riga, Latvia). The data were calculated in relation to the untreated control (100 %).

### Statistical analysis

Statistical methods were used to interpret the results and draw conclusions from the experimental data, considering both univariate and multivariate approaches (*25*). We used the Statistica v. 13.3.1 programme (*26*). All experiments were performed in triplicate to ensure the reliability and reproducibility of the results. A one-way analysis of variance (ANOVA) was done to compare the mean values of the different samples. Duncan's new multiple range test (DNMRT) was used for *post-hoc* analysis, *i.e*. to determine which groups differed significantly from each other after the ANOVA. The results were considered statistically significant if the p-value was less than or equal to 0.05 (p≤0.05). Principal component (PC) analysis was performed to reduce dimensionality and visualise complex data sets, and highlight patterns and relationships in the data. Pearson’s correlation coefficients were calculated to quantify the linear relationship between phytochemical content, antioxidant capacity and species bioactivities.

## RESULTS AND DISCUSSION

### RP-HPLC qualitative and quantitative characterisation of flavonoids

**Fig. S1:**
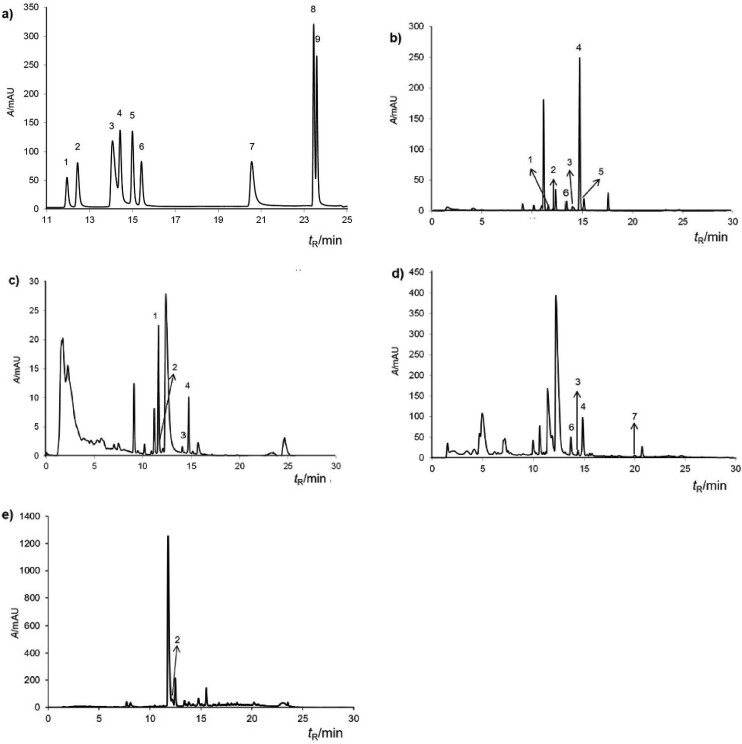
RP-HPLC profiles recorded at *λ*=360 nm of: a) phenolic standards, and phenolic compounds from non-hydrolysed aqueous infusions of b) *Calendula officinalis* L., c) *Chelidonium majus* L., d) *Alchemilla vulgaris* L., and e) *Teucrium chamaedrys* L. 1=quercetin-3-*O*-rutinoside, 2=quercetin-3-β-d-glucoside, 3=kaempferol-3-*O*-rutinoside, 4=isorhamnetin-3-*O*-rutinoside, 5=isorhamnetin-3-*O*-glucoside, 6=quercetagetin, 7=quercetin, 8=kaempferol, 9=isorhamnetin

A new RP-HPLC method was developed to achieve optimal separation of flavonoids in all tested extracts. The results of the analysis of non-hydrolysed infusions compared to the standard are shown in Fig. S1. The chromatogram of the standard flavonoid compounds is shown in Fig. S1a, while the peak designations, calibration curves and R^2^ values are listed in Table S1. The chromatogram of the non-hydrolysed infusion of *C. officinalis* is shown in Fig. S1b. The predominant peak was identified as isorhamnetin-3-*O*-rutinoside (4), followed by isorhamnetin-3-*O*-glucoside (5) and quercetagetin (6). A lower peak was identified as quercetin-3-*O*-rutinoside (1) and only trace amounts of quercetin-3-β-d-glucoside (2) and kaempferol-3-*O*-rutinoside (3) were present. Isorhamnetin-3-*O*-rutinoside was present on dry mass basis at 8.34 mg/kg.

The RP-HPLC chromatogram of the non-hydrolysed infusion of *C. majus* is shown in Fig. S1c. The second most significant peak was identified as quercetin-3-*O*-rutinoside (1) on dry mass basis at 2.17 mg/kg, followed by the peak identified as isorhamnetin-3-*O*-rutinoside (4). Among the identified flavonoid glycosides, quercetin-3-*O*-rutinoside accounted for 75.1 %. Only small amounts of quercetin-3-β-d-glucoside (2) and kaempferol-3-*O*-rutinoside (3) were detected. Quercetin was also found as the predominant flavonoid aglycone in a *C. majus* extract from Poland, whereas isorhamnetin was not identified at all (*27*). Quercetin-3-*O*-rutinoside as the predominant compound and isorhamnetin-3-*O*-rutinoside as the second most abundant flavonol glycoside were detected in extracts of wild and cultivated *C. majus* (*28*). Fig. S1d shows the RP-HPLC profile of glycosides from the aqueous infusion of *A. vulgaris*. The main constituent of *A. vulgaris* was isorhamnetin-3-*O*-rutinoside (4). The second most abundant glycoside was kaempferol-3-*O*-rutinoside (3). The RP-HPLC chromatogram of the *T. chamaedrys* infusion is shown in Fig. S1e. Only one flavonoid glycoside was identified, namely quercetin-3-β-d-glucoside (2).

**Fig. 1:**
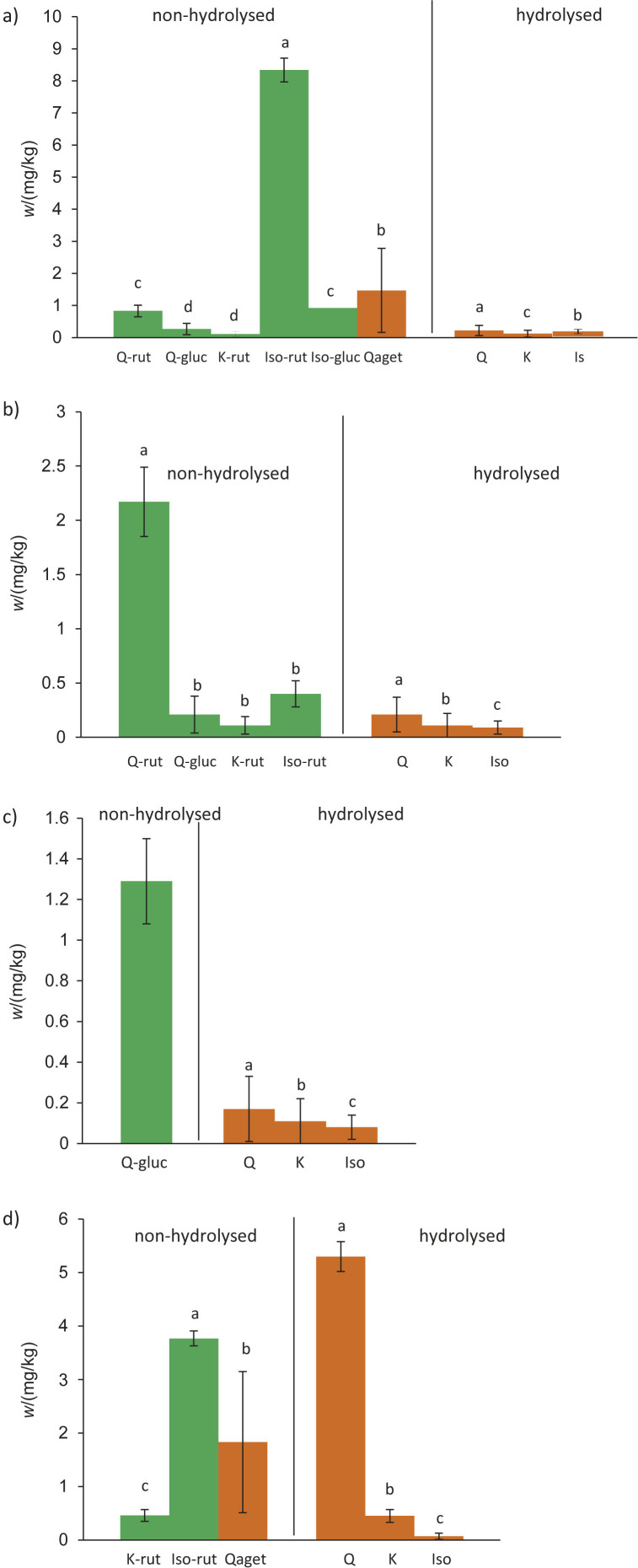
Composition of the main flavonoids in aqueous infusions (non-hydrolysed and hydrolysed) of: a) *Calendula officinalis* L., b) *Chelidonium majus* L., c) *Teucrium chamaedrys* and d) *Alchemilla vulgaris* L. Data are the mean values±S.D, *N*=3. Q=quercetin, Qaget=quercetagetin, K=kaempferol, Iso=isorhamnetin, Q-rut=quercetin-3-*O*-rutinoside, Q-gluc=quercetin-3-*O*-glucoside, K-rut=kaempferol-3-*O*-rutinoside, Iso-rut=isorhamnetin-3-*O*-rutinoside, Iso-gluc=isorhamnetin-3-*O*-glucoside, DM=dry mass. Green bars correspond to flavonoid glycosides, orange bars to flavonoid aglycones. The values represent the mean value±S.D., *N*=3. Different letters indicate a significant difference among the values (ANOVA, Duncan's test, p≤0.05). Statistics was done separately for non-hydrolysed and separately for hydrolysed samples

Isorhamnetin-3-*O*-rutinoside accounted for 69.9 % of the total flavonoid glycosides identified in the aqueous infusion of *C. officinalis* (Fig. 1a). The mass fraction on dry mass basis of the second most abundant glycoside (isorhamnetin-3-*O*-glucoside) was 0.92 mg/kg of *C. officinalis*, while for quercetin-3-*O*-rutinoside, quercetin-3-*O*-glucoside and aglycone quercetagetin it was 0.83, 0.27 and 1.47 mg/kg, respectively. After the acid hydrolysis, mass fraction of quercetin was the highest among the identified aglycones (Fig. 1a).

Compared to the flavonoid glycosides detected in the 70 % methanolic extract of *C. officinalis* from Tunisia (*29*), we found two additional glycosides in the water extract of Croatian *C. officinalis* – isorhamnetin-3-*O*-rutinoside (which was predominant) and kaempferol-3-*O*-rutinoside. The most likely reason for this difference is the use of different extraction solvents and a completely different collection area. In addition, plants can be collected and processed in different ways and in different forms. This is because the analysed samples were raw materials collected in different areas and non-standardised purchased products. Interestingly, ethanolic extracts of *C. officinalis* inflorescences from France, Egypt and Russia contained quercetin and isorhamnetin glycosides, but no kaempferol glycosides were detected (*30*-*32*). So far, the presence of kaempferol-3-*O*-glucoside and kaempferol has been detected in *C. officinalis*, but we have not found kaempferol-3-*O*-rutinoside in Croatian species (*11*). Therefore, to the best of our knowledge, this is the first report of the detection of kaempferol-3-*O*-rutinoside in the aqueous infusion of *C. officinalis*. In *C. majus*, quercetin-3-*O*-rutinoside, quercetin-3-β-d-glucoside, kaempferol-3-*O*-rutinoside and isorhamnetin-3-*O*-rutinoside were tentatively identified (Fig. 1b). The main constituent of *T. chamaedrys* was quercetin glucoside (Fig. 1c), while in *A. vulgaris* it was isorhamnetin-3-*O*-rutinoside, which accounted for 89.1 % of the identified flavonoid glycosides (Fig. 1d).

**Fig. S2:**
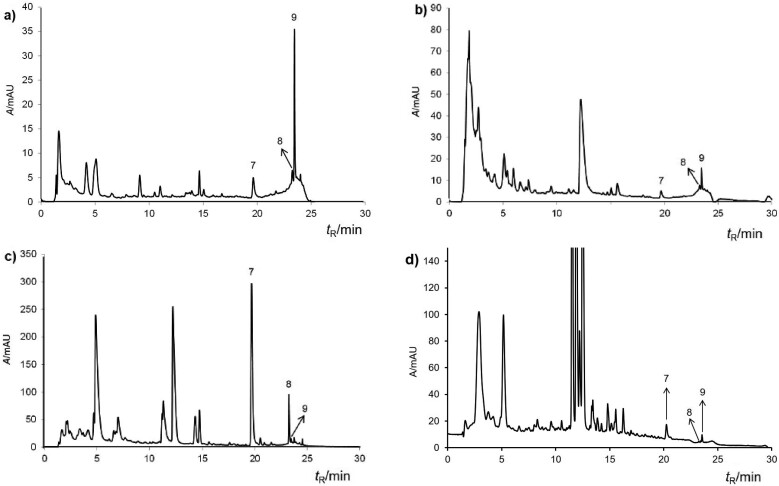
RP-HPLC profiles recorded at *λ*=360 nm of phenolics from hydrolysed aqueous infusions of: a) *Calendula officinalis* L., b) *Chelidonium majus* L., c) *Alchemilla vulgaris* L. and d) *Teucrium chamaedrys* L. 7=quercetin, 8=kaempferol, 9=isorhamnetin

Acid hydrolysis of the aqueous infusion of *C. officinalis* released free aglycones (Fig. S2a). Although isorhamnetin (9) showed the highest peak in the chromatogram (Fig. S2a), among the aglycones in the hydrolysed extract, the mass fraction of quercetin (7) was the highest (Fig. 1a). After acid hydrolysis of *C. majus*, the peaks of free quercetin (7), kaempferol (8) and isorhamnetin (9) were detected (Fig. S2b). Quercetin glycoside was present at the highest mass fraction (Fig. 1b). As in the non-hydrolysed infusion of *C. officinalis* (Fig. S1b), the non-hydrolysed extract of *A. vulgaris* also contained the aglycone quercetagetin (6) (Fig. S1d). Interestingly, after acid hydrolysis, quercetin (7) was found to be the predominant component (Fig. S2c), suggesting that one or more of the glycosylated peaks in this extract correspond to quercetin glycosides, although they were not identified. Many quercetin glycosides have been found in various *Alchemilla* species from Turkey, which also supports our conclusion (*33*, *34*). In addition, the flavonoids of *Alchemilla* species are usually quercetin derivatives (*34*). Our finding of isorhamnetin-3-*O*-rutinoside is, as far as we know, the first report of this flavonoid in *A. vulgaris*. After acid hydrolysis of *T. chamaedrys*, lower peaks corresponding to free quercetin (7), kaempferol (8) and isorhamnetin (9) were detected (Fig. S2d). This indicates that kaempferol and isorhamnetin glycoside forms were present in the non-hydrolysed extract, which differ from those available to us as standards.

**Fig. S3:**
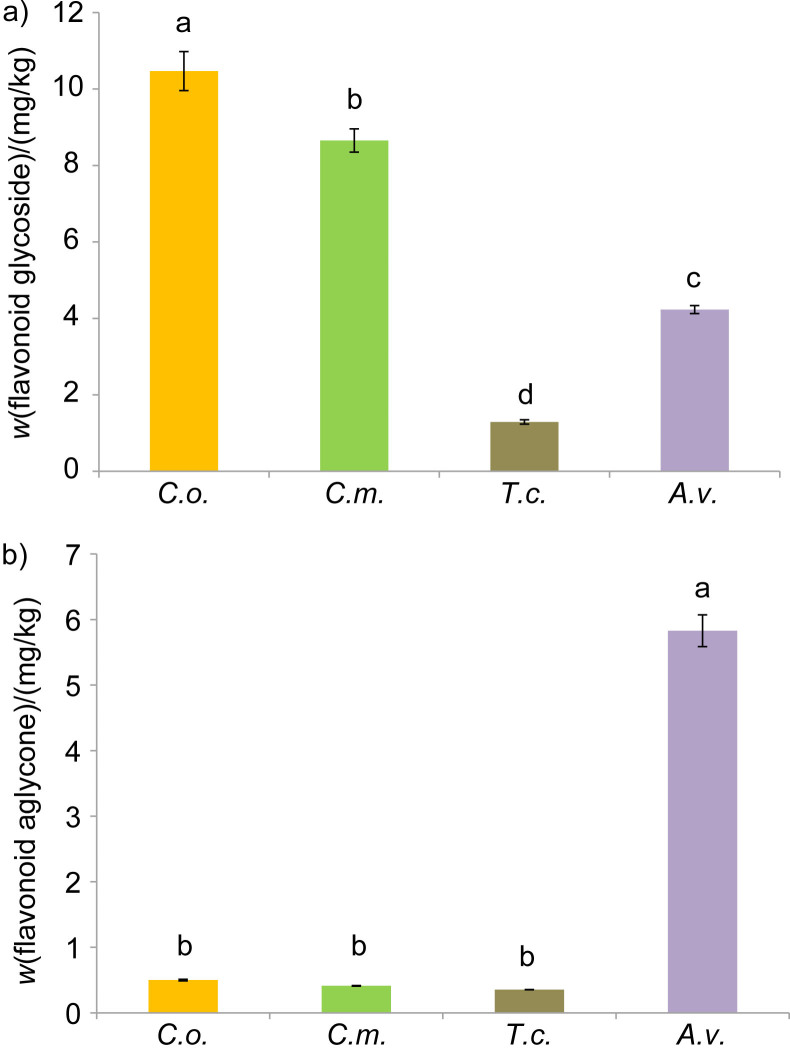
Mass fractions of identified flavonoids on dry mass basis: a) glycosydes in non-hydrolysed extracts, and b) aglycones in hydrolysed extracts of *Calendula officinalis* L. (*C.o.*), *Chelidonium majus* L. (*C. m.*), *Teucrium chamaedrys* (*T.c.*) and *Alchemilla vulgaris* L. (*A.v.*) shoots. Data are the mean values of three replicates±S.D. Different letters indicate a significant difference among the values (ANOVA, Duncan's test, p≤0.05)

After summarising all identified glycosides in non-hydrolysed extracts, their mass fraction was the highest in *C. officinalis* (Fig. S3a), while after hydrolysis the highest mass fraction of identified aglycones was found in *A. vulgaris* (Fig. S3b). According to the available data, there is no information so far on the presence of phenolic compounds in the aqueous infusion of *T. chamaedrys*.

### Total phenolic content and biological activity of plant infusions

Table 1 shows the total phenolic content and antioxidant activity of aqueous infusions of selected medicinal plants. The highest total phenolic content (expressed as GAE) was found in the infusion of *T. chamaedrys* ((2061±42) mg/L), while the infusion of *C. officinalis* had the lowest total phenolic content ((638±35) mg/L), which was 30 % lower than that of *T. chamaedrys*. The results are in agreement with those of Vlase *et al*. (*35*), who reported that the extract of *T. chamaedrys* contained the highest amount of flavonoid compounds compared to the extracts of *Hyssopus officinalis* and *Ocimum basilicum*. The highest free radical scavenging activity was found in *A. vulgaris* infusion (IC_50_=0.8 mg/mL) using DPPH assay and in *T. chamaedrys* infusion (9798 mg/L, expressed as FeCl_2_) using FRAP assay (Table 1). *C. officinalis* had the lowest antioxidant activity and the lowest concentration of total phenolics in all used methods. High antioxidant activity and lipid inhibition potential of the methanolic extract of *A. vulgaris* have been reported (*17*, *18*). Özer (*36*) demonstrated a high antioxidant potential of the infusion and decoction of *T. chamaedrys* using DPPH and Cu(II) reducing antioxidant capacity (CUPRAC) assays. The ethanol extract of *T. chamaedrys* also showed a significant free radical scavenging activity (*35*). According to the available data, there were no reports so far on the influence of extracts of plants used in this study on the oxidative stability of edible oils measured by the Rancimat method. The protection factor (PF) of *C*. majus was significantly higher (1.19±0.03) than that of the other tested infusions (Table 1) and was similar to that of α-tocopherol, a commercially available antioxidant (*37*), so it was concluded that it could be used to preserve the oxidative stability of edible oils. The ability of medicinal plants to prolong the oxidative stability and shelf life of foods containing oil is important for the food industry and pharmaceutical sector. However, herbs and plants can be processed using different techniques and ingested in different ways and forms. Although this aspect was not the focus of this study, it may be interesting to conduct a comparative study on the use of different processing techniques of selected medicinal herbs to protect their biological activity.

**Table 1:** Total phenolic content and antioxidant potential of aqueous infusions of selected medicinal plants

Sample	Total phenols as *γ*(GAE)/(mg/L)	DPPH scavenging as IC_50_/(mg/mL)	FRAP as *γ*(FeCl_2_)/(mg/L)	Rancimat PF
*Calendula officinalis* L.	(638±35)^d^	nd	(425±18)^d^	nd
*Chelidonium majus* L.	(1123±63)^c^	(5.16±0.01)^a^	(1377±32)^c^	(1.19±0.03)^a^
*Teucrium chamaedrys* L.	(2061±42)^a^	(1.90±0.02)^b^	(9798±27)^a^	(1.12±0.01)^b^
*Alchemilla vulgaris* L.	(1267±24)^b^	(0.80±0.01)^c^	(6952±20)^b^	(1.08±0.01)^b^
BHT	/	(0.018±0.002)	/	(3.6±0.2)
BHA	/	(0.054±0.002)	/	(7.2±0.1)
Ascorbic acid	/	/	(2.60±0.22)·10^-4^	/

Values represent mean value±S.D., *N*=3. Different letters indicate a significant difference among the values in a column (ANOVA, Duncan’s test, p≤0.05). GAE=gallic acid equivalents, PF=protection factor, BHT=butylated hydroxytoluene, BHA=butylated hydroxyanisole, nd=not determined, /=not measured

The antiproliferative activity of the infusions of the selected medicinal plants tested against the cancer cell lines MD-MBA-231, T24 and A549 is shown in Table 2, Table 3 and Table 4 respectively in relation to the dose-dependent effect (1, 0.5, 0.25 and 0.1 mg/mL) and different incubation times (4, 24, 48 and 72 h). At a concentration of 1 mg/mL, *A. vulgaris* showed the highest inhibition of MDA-MD-231 cell proliferation at each time point (Table 2). At the same concentration, the inhibition rate of cancer cell proliferation by infusion of *A. vulgaris* was as high as 75 % after 72 h. At lower concentrations, *A. vulgaris* had the highest inhibition rate after 4 h, at later time points it was *C. majus*. The inhibition rate of *T. chamaedrys* infusion was below the detection limit at 0.50 mg/mL. The aqueous infusion of *C. officinalis* had a low antiproliferative effect against MDA-MD-231 cells (Table 2). *C. majus* aqueous infusion showed a high and dose-dependent antiproliferative effect against MDA-MD-231 cells (Table 2), which is consistent with other studies (*38*, *39*). According to the available data, this is the first comparative report on the antiproliferative effect of *A. vulgaris* and *C. majus* infusions against MDA-MD-231 cells.

**Table 2:** *In vitro* antiproliferative activity of aqueous infusions of selected plants at different concentrations against MD-MBA-231 cancer cells

*γ*(sample)/(g/L)	Inhibition of cell proliferation/%			
1.00	*t*(incubation)/h			
	4	24	48	72
*Calendula officinalis* L.	(3.60±0.02)^c^	(8.86±0.02)^c^	(19.60±0.06)^d^	(11.51±0.05)^d^
*Chelidonium majus* L.	(15.85±0.03)^b^	(38.96±0.03)^b^	(54.40±0.01)^b^	(61.95±0.02)^b^
*Teucrium chamaedrys* L.	(0.00±0.00)^d^	(0.00±0.00)^d^	(25.60±0.04)^c^	(39.53±0.02)^c^
*Alchemilla vulgaris* L.	(22.70±0.01)^a^	(51.81±0.03)^a^	(68.00±0.04)^a^	(74.93±0.05)^a^
0.50				
*Calendula officinalis* L.	(0.00±0.00)^c^	(3.64±0.03)^c^	(17.10±0.06)^c^	(10.90±0.03)^c^
*Chelidonium majus* L.	(15.70±0.03)^b^	(34.76±0.01)^a^	(44.11±0.02)^a^	(58.40±0.04)^a^
*Teucrium chamaedrys* L.	/	/	/	/
*Alchemilla vulgaris* L.	(22.10±0.05)^a^	(34.43±0.03)^b^	(37.01±0.02)^b^	(45.70±0.04)^b^
0.25				
*Calendula officinalis* L.	(0.00±0.00)^c^	(3.25±0.03)^c^	(14.02±0.03)^c^	(2.60±0.04)^c^
*Chelidonium majus* L.	(11.70±0.03)^b^	(28.86±0.03)^a^	(42.00±0.02)^a^	(51.60±0.03)^a^
*Teucrium chamaedrys* L.	/	/	/	/
*Alchemilla vulgaris* L.	(17.44±0.01)^a^	(25.12±0.03)^b^	(32.12±0.03)^b^	(41.50±0.03)^b^
0.10				
*Calendula officinalis* L.	(0.00±0.00)^b^	(0.00±0.00)^c^	(7.10±0.03)^c^	(0.00±0.00)^c^
*Chelidonium majus* L.	(9.97±0.03)^a^	(26.23±0.04)^a^	(32.12±0.02)^a^	(46.60±0.03)^a^
*Teucrium chamaedrys* L.	/	/	/	/
*Alchemilla vulgaris* L.	(14.5±3.3)^a^	(20.0±4.2)^b^	(21.40±0.03)^b^	(33.42±8.07)^b^
Positive control (*γ*(cisplatin)=50 µg/mL)	(13.6±2.3)	(20.7±1.8)	(50.2±1.0)	(79.4±1.5)

Values represent mean value±S.D., *N*=3. Different letters in superscript indicate a significant difference among the values in the same column (ANOVA, Duncan’s test, p≤0.05). /=not measured

**Table 3:** *In vitro* antiproliferative activity of aqueous infusions of selected plants at different concentrations against T-24 cancer cells

*γ*(sample)/(g/L)	Inhibition of cell proliferation/%						
	*t*(incubation)/h						
1.00	4		24		48		72
*Calendula officinalis* L.	(15.16±0.02)^a^	(39.45±0.04)^b^		(58.29±0.05)^b^		(78.17±0.03)^b^	
*Chelidonium majus* L.	(10.75±0.03)^c^	(41.49±0.02)^a^		(86.62±0.03)^a^		(96.25±0.03)^a^	
*Teucrium chamaedrys* L.	(0.00±0.00)^d^	(5.54±0.03)^d^		(32.52±0.03)^d^		(13.85±0.06)^d^	
*Alchemilla vulgaris* L.	(13.36±0.02)^b^	(21.30±0.03)^c^		(51.69±0.01)^c^		(62.92±0.03)^c^	
0.50							
*Calendula officinalis* L.	(0.00±0.00)^c^	(27.36±0.02)^b^		(28.53±0.03)^c^		(28.77±0.03)^c^	
*Chelidonium majus* L.	(11.89±0.06)^b^	(30.88±0.06)^a^		(51.39±0.00)^a^		(79.36±0.06)^a^	
*Teucrium chamaedrys* L.	nd	nd		nd		nd	
*Alchemilla vulgaris* L.	(13.85±0.03)^a^	(20.10±0.06)^c^		(47.08±0.03)^b^		(48.57±0.02)^b^	
0.25							
*Calendula officinalis* L.	(14.01±0.03)^b^	(25.28±0.03)^b^		(20.25±0.01)^c^		(28.43±0.03)^c^	
*Chelidonium majus* L.	(2.45±0.03)^c^	(26.72±0.03)^a^		(44.33±0.01)^a^		(53.81±0.01)^a^	
*Teucrium chamaedrys* L.	nd	nd		nd		nd	
*Alchemilla vulgaris* L.	(14.47±0.03)^a^	(17.20±0.02)^c^		(40.24±0.01)^b^		(37.10±0.02)^b^	
0.10							
*Calendula officinalis* L.	(6.85±0.03)^a^	(21.23±0.01)^a^		(9.51±0.03)^c^		(24.37±0.05)^c^	
*Chelidonium majus* L.	(0.00±0.00)^b^	(14.22±0.03)^c^		(17.64±0.03)^b^		(38.20±0.02)^a^	
*Teucrium chamaedrys* L.	nd	nd		nd		nd	
*Alchemilla vulgaris* L.	(0.00±0.00)^b^	(15.84±0.03)^b^		(30.40±0.04)^a^		(26.40±0.03)^b^	
Positive control (*γ*(cisplatin)=50 µg/mL)	(8.4±1.2)	(13.7±2.2)		(43.8±1.6)		(47.69±0.09)	

Values represent mean value±S.D., *N*=3. Different letters in superscript indicate a significant difference among the values in the same column (ANOVA, Duncan’s test, p≤0.05)

**Table 4:** *In vitro* antiproliferative activity of aqueous infusions of selected plants at different concentrations against A549 cancer cells

γ(sample)/(g/L)		Inhibition of cell proliferation/%									
		*t*(incubation)/h									
1.00		4			24			48			72
*Calendula officinalis* L.		(9.52±0.02)^b^	(14.29±0.04)^d^			(16.48±0.05)^d^			(21.43±0.03)^d^		
*Chelidonium majus* L.		(20.31±0.04)^a^	(32.00±0.05)^a^			(41.43±0.04)^b^			(51.30±0.01)^a^		
*Teucrium chamaedrys* L.		(3.01±0.01)^d^	(20.95±0.02)^c^			(40.82±0.05)^c^			(36.97±0.01)^c^		
*Alchemilla vulgaris* L.		(7.13±0.04)^c^	(21.18±0.03)^b^			(43.27±0.04)^a^			(45.54±0.01)^b^		
0.50											
*Calendula officinalis* L.		(0.00±0.00)	(15.20±0.05)^c^			(20.10±0.03)^c^			(11.20±0.03)^c^		
*Chelidonium majus* L.		(7.10±0.04)^a^	(20.10±0.01)^a^			(31.20±0.04)^b^			(40.0±0.0)^b^		
*Teucrium chamaedrys* L.		/	/			/			/		
*Alchemilla vulgaris* L.		(6.21±0.01)^a^	(16.73±0.02)^b^			(41.59±0.06)^a^			(48.04±0.03)^a^		
0.25											
*Calendula officinalis* L.		(1.20±0.03)^b^	(8.10±0.06)^c^			(19.20±0.03)^b^			(11.00±0.03)^c^		
*Chelidonium majus* L.		(6.50±0.03)^a^	(17.40±0.03)^a^			(30.00±0.02)^a^			(34.70±0.04)^a^		
*Teucrium chamaedrys* L.		/	/			/			/		
*Alchemilla vulgaris* L.		(0.00±0.00)^c^	(14.22±0.02)^b^			(16.04±0.04)^c^			(26.79±0.03)^b^		
0.10											
*Calendula officinalis* L.		(0.00±0.00)	(9.20±0.03)^b^			(15.00±0.02)^b^			(6.50±0.04)^c^		
*Chelidonium majus* L.		(0.00±0.00)	(9.20±0.02)^b^			(29.10±0.03)^a^			(32.10±0.03)^a^		
*Teucrium chamaedrys* L.		/	/			/			/		
*Alchemilla vulgaris* L.		(0.00±0.00)	(11.02±0.06)^a^			(12.48±0.03)^c^			(9.47±0.06)^b^		
Positive control (*γ*(cisplatin)=50 µg/mL)		(0.00±0.00)	(8.0±1.2)			(17.7±1.8)			(15.3±0.1)		

Values represent mean value±S.D., *N*=3. Different letters in superscript indicate a significant difference among the values in the same column (ANOVA, Duncan’s test, p≤0.05). /=not measured

The antiproliferative activity against T24 cancer cells was the highest when using *C. majus* infusion (Table 3). At a concentration of 1 mg/mL, the antiproliferative activity of *C. majus* was as high as 96 % after 72 h of incubation. The antiproliferative activity of *T. chamaedrys* was already low at a concentration of 1 mg/mL, and at lower concentrations it was below the detection limit (data not shown). To the best of our knowledge, this is the first study on the antiproliferative activity of selected plant infusions against T24 cancer cells.

The antiproliferative activity of the infusions was lower in cancer cells A549 than in cancer cells MDA-MB-231 and T24 (Table 4). The antiproliferative activity of 1 mg/mL of *C. officinalis* and *T. chamaedrys* against A549 cells after 72 h incubation was low, while the activity of *C. majus* and *A. vulgaris* was moderate (51 and 46 %, respectively). The alcoholic extract of *T. chamaedrys* did not show any antiproliferative activity against A549 cells (*40*). According to the available data (*38*), the antiproliferative activity of the extract of *C. majus* against A549 cells was moderate, which is consistent with our results. To the best of our knowledge, there are no previous data on the comparative report of the antiproliferative activity of *T. chamaedrys* and *A. vulgaris* against A549 cells.

### Results of statistical analysis

**Fig. 2:**
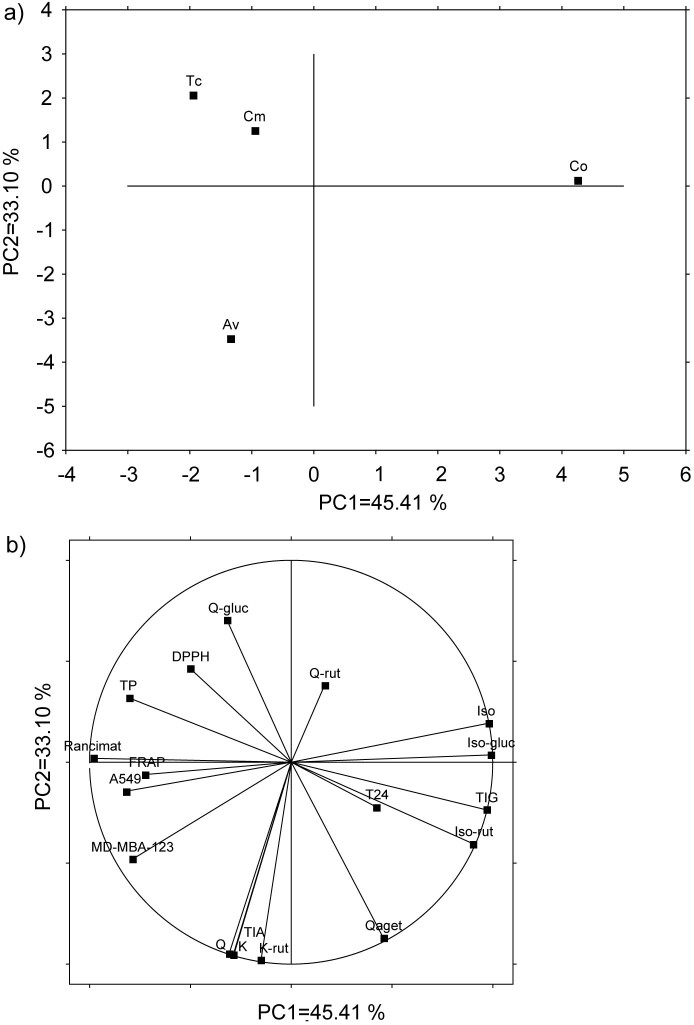
Principal component (PC) analysis of total phenolics, individual identified phenolic compounds, antioxidant capacity and antiproliferative activity of herbal infusions: a) score plot separating the samples based on the measured variables, and b) loading plot of the measured variables. Co=*Calendula officinalis*, Cm=*Chelidonium majus*, Tc=*Teucrium chamaedrys*, Av*=Alchemilla vulgaris*, TP=total phenolics, FRAP=Fe(III) reducing antioxidant power, DPPH=2,2-diphenyl-1-picrylhydrazyl, Rancimat=effect on the oil oxidative stability, Q=quercetin, Qaget=quercetagetin, K=kaempferol, Iso=isorhamnetin, Q-rut=quercetin-3-*O*-rutinoside, Q-gluc=quercetin-3-*O*-glucoside, K-rut=kaempferol-3-*O*-rutinoside, Iso-rut=isorhamnetin-3-*O*-rutinoside, Iso-gluc=isorhamnetin-3-*O*-glucoside, TIG=total identified glucosides before hydrolysis, TIA=total identified aglycones after hydrolysis, MD-MBA-123=breast cancer cells, A549=lung cancer cells, T24=urinary bladder cancer cells

Based on the principal component (PC) analysis of the measured parameters, *T. chamaedrys* and *C. majus* were grouped together, meaning these were the most similar infusions, while *A. vulgaris* and *C. officinalis* were more distant from this group as well as from each other (Fig. 2a). The loading plot of the measured variables showed that quercetin glucoside and antioxidant capacity measured by the DPPH method contributed most to the separation of these infusions from the two others (Fig. 2b). The main contributors to the separation of *A. vulgaris* were quercetin, kaempferol, kaempferol-3-*O*-rutinoside, total identified aglycones and the rate of inhibition of proliferation of MD-MBA-231. The separation of *C. officinalis* was mainly due to the total identified glucosides, isorhamnetin, isorhamnetin-3-*O*-rutinoside and isorhamnetin-3-*O*-glucoside (Fig. 2b).

**Fig. 3:**
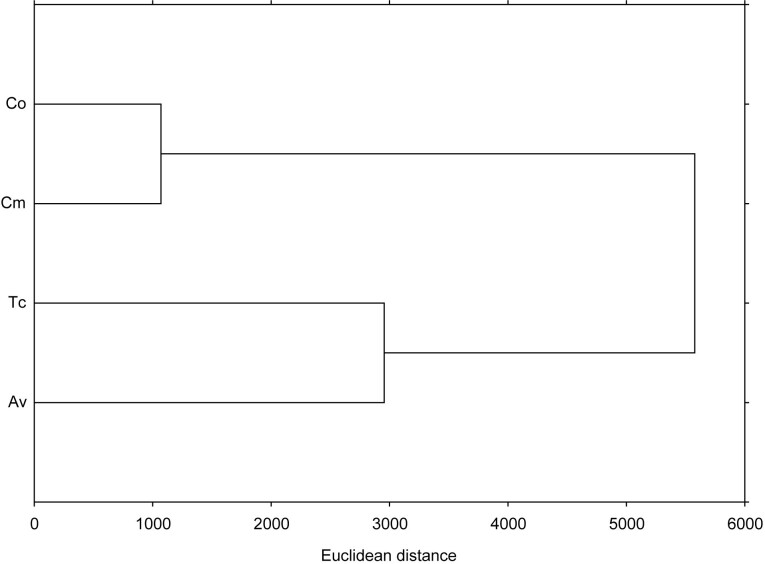
Dendrogram based on the single linkage method of clustering between *Calendula officinalis*, *Chelidonium majus*, *Teucrium chamaedrys* and *Alchemilla vulgaris* calculated for all the measured parameters. The measuring unit is Euclidean distance

Hierarchical clustering shows the relationships between different data sets and indicates the degree of similarity/dissimilarity between samples. Based on the total amount and individual phenolics identified, antioxidant capacity and cytotoxicity, *C. officinalis* and *C. majus* were the least distant from each other and formed a cluster (Fig. 3). *T. chamaedrys* and *A. vulgaris* formed another cluster, but were more distant from each other than *C. officinalis* and *C. majus*.

According to Evans (*41*), Pearson’s correlation coefficient (r) between the measured parameters of the samples showed a very strong positive correlation between the antioxidant capacity (FRAP) and total phenolics (Table S2), between the antioxidant capacity measured with the Rancimat method and the inhibition rate of MD-MBA-123 and A549 cells, and between the inhibition rate of MD-MBA-123 and A549 cells.

## CONCLUSIONS

The focus of this study was the comparative analysis of the main flavonoids and biological potential (antioxidant and antiproliferative) of aqueous infusions from four medicinal plants: *Calendula officinalis* L., *Chelidonium majus* L., *Teucrium chamaedrys* L. and *Alchemilla vulgaris* L. Using the newly developed RP-HPLC method for optimal separation, tentative identification and quantification of flavonoid glycosides and aglycones, the presence of kaempferol-3-*O*-rutinoside in *C. officinalis* and isorhamnetin-3-*O*-rutinoside in *A. vulgaris* aqueous infusions was reported for the first time. A comparative analysis of the main flavonoids revealed that isorhamnetin-3-*O*-rutinoside was found in the aqueous infusion of *C. officinalis* (69.9 % of the total flavonoid glycosides), quercetin-3-*O*-rutinoside in the infusion of *C. majus* (75.1 % of the total flavonoid glycosides), isorhamnetin-3-*O*-rutinoside in the infusion of *A. vulgaris* (89.1 % of the total flavonoid glycosides) and quercetin-3-β-d-glucoside in the infusion of *T. chamaedrys* as the dominant flavonoids. *T. chamaedrys* had the highest values of total phenolics, free radical scavenging activity and Fe(III) reducing antioxidant power. *C. majus* contributed to prolonging the oxidative stability of edible oil. This is the first report on the antiproliferative activity of *A. vulgaris* and *C. majus* infusions against MDA-MD-231 cells, of *T. chamaedrys* and *A. vulgaris* infusions against A549 cells, and of all prepared aqueous infusions against T24 cells. A very high antiproliferative activity was obtained by the infusion of *C. majus* against T24 cells (96 % at a concentration of 1 mg/mL after 72 h of incubation) and *A. vulgaris* infusion against MDA-MD-231 cells (75 % at 1 mg/mL after 72 h of incubation). Overall, this comparative study contributes to the knowledge of the powerful health benefits of medicinal plants and their role in the food and pharmaceutical industries, as well as the potential use of natural products in medicine. Investigating the effects of different processing methods (extraction, drying, freezing, grinding) on the protection of the main biologically active compounds in the plant material (whole or cut, raw or dried) remains a challenge for scientists and producers.

## Appendix

**Table S1:** Peak designations, calibration curves and R*^2^* values of flavonoid standards

Peak	Phenolic compound	Calibration curve	R^2^
1	quercetin-3-*O*-rutinoside	y=451.72x-13.644	0.997
2	quercetin-3-β-*D*-glucoside	y=823.37x-25.617	0.998
3	kaempferol-3-*O*-rutinoside	y=1032.1x-15.212	0.999
4	isorhamnetin-3-*O*-rutinoside	y=1126x-18.995	0.999
5	isorhamnetin-3-*O*-glucoside	y=778.99x+2.4566	1.000
6	quercetagetin	y=2703.3x-634.05	0.992
7	quercetin	y=866.26x-83.147	0.987
8	kaempferol	y=1730.8x-108.87	0.997
9	isorhamnetin	y=1873.5x-63.697	0.998
Data are the mean values of *N*=3. Test of linearity range was conducted at *γ*=1–250 µg/mL, except for isorhamnetin where it was *γ*=0.7–167 µg/mL			

**Table S2:** Pearson’s correlation coefficients (r) between the phenolics, antioxidant and antiproliferative activity of aqueous infusions of *Calendula officinalis*, *Chelidonium majus*, *Teucrium chamaedrys* and *Alchemilla vulgaris*

Factor	TP	DPPH	FRAP	Rancimat	Q-rut	Q-gluc	K-rut	Iso-rut	Iso-gluc	Q	K	Iso	Qaget	TIG	TIA	MD-MBA-231	T24	A549
TP	1.000																	
DPPH	0.180	1.000																
FRAP	**0.908**	-0.169	1.000															
Rancimat	0.697	0.641	0.577	1.000														
Q-rut	-0.469	0.761	-0.767	0.023	1.000													
Q-gluc	0.799	0.016	0.624	0.202	-0.365	1.000												
K-rut	-0.250	-0.294	0.090	0.151	-0.293	-0.715	1.000											
Iso-rut	-0.784	-0.742	-0.533	**-0.921**	-0.134	-0.481	0.245	1.000										
Iso-gluc	-0.716	-0.578	-0.626	**-0.997**	0.052	-0.203	-0.200	**0.899**	1.000									
Q	-0.014	-0.343	0.336	0.268	-0.483	-0.517	**0.968**	0.117	-0.328	1.000								
K	-0.027	-0.363	0.329	0.246	-0.491	-0.521	**0.969**	0.139	-0.307	**1.000**	1.000							
Iso	-0.721	-0.409	-0.715	**-0.961**	0.224	-0.169	-0.339	**0.810**	**0.980**	-0.484	-0.465	1.000						
Qaget	-0.551	-0.766	-0.151	-0.500	-0.424	-0.639	0.764	0.791	0.446	0.699	0.715	0.282	1.000					
TIG	**-0.853**	-0.599	-0.686	**-0.959**	0.064	-0.471	0.086	**0.974**	**0.954**	-0.074	-0.053	**0.908**	0.651	1.000				
TIA	-0.036	-0.356	0.319	0.246	-0.480	-0.531	**0.972**	0.140	-0.306	**1.000**	**1.000**	-0.463	0.715	-0.050	1.000			
MD-MBA-231	0.289	0.452	0.339	**0.839**	0.036	-0.331	0.635	-0.572	**-0.851**	0.669	0.651	**-0.871**	-0.012	-0.655	0.655	1.000		
T24	**-0.852**	0.331	**-0.895**	-0.247	0.785	**-0.850**	0.256	0.346	0.290	0.010	0.011	0.361	0.223	0.457	0.023	0.110	1.000	
A549	0.333	0.750	0.221	**0.908**	0.317	-0.195	0.328	-0.760	**-0.892**	0.344	0.322	-0.837	-0.352	-0.765	0.327	**0.927**	0.175	1.000
